# Pneumococcal capsule blocks protection by immunization with conserved surface proteins

**DOI:** 10.1038/s41541-021-00413-5

**Published:** 2021-12-20

**Authors:** Tonia Zangari, M. Ammar Zafar, John A. Lees, Annie R. Abruzzo, Gavyn Chern Wei Bee, Jeffrey N. Weiser

**Affiliations:** 1 Department of Microbiology, New York University Grossman School of Medicine, New York, NY USA; 2 Present Address: Department of Microbiology and Immunology, Wake Forest School of Medicine, Winston-Salem, NC USA; 3 Present Address: Department of Infectious Disease Epidemiology, Medical Research Council Centre for Global Infectious Disease Analysis, Imperial College London, London, UK

**Keywords:** Protein vaccines, Bacterial infection

## Abstract

Vaccines targeting *Streptococcus pneumoniae* (Spn) are limited by dependence on capsular polysaccharide and its serotype diversity. More broadly-based approaches using common protein antigens have not resulted in a licensed vaccine. Herein, we used an unbiased, genome-wide approach to find novel vaccine antigens to disrupt carriage modeled in mice. A Tn-Seq screen identified 198 genes required for colonization of which 16 are known to express conserved, immunogenic surface proteins. After testing defined mutants for impaired colonization of infant and adult mice, 5 validated candidates (StkP, PenA/Pbp2a, PgdA, HtrA, and LytD/Pce/CbpE) were used as immunogens. Despite induction of antibody recognizing the Spn cell surface, there was no protection against Spn colonization. There was, however, protection against an unencapsulated Spn mutant. This result correlated with increased antibody binding to the bacterial surface in the absence of capsule. Our findings demonstrate how the pneumococcal capsule interferes with mucosal protection by antibody to common protein targets.

## Introduction

*Streptococcus pneumoniae* (Spn, the pneumococcus) is a common member of the human upper respiratory tract (URT) flora, found in 25–65% of children and <7% of adults^1^. Access by the organism to normally sterile sites can result in inflammatory diseases such as otitis media, community-acquired pneumonia, and bacterial meningitis. Because of the high burden of disease and increasing resistance to many common antimicrobial agents, the World Health Organization included Spn as one of its 12 priority pathogens.

Capsular polysaccharide-based conjugate vaccines (PCV) have reduced the incidence of invasive Spn disease for included serotypes^2^. An unexpected benefit of PCV is decreased acquisition of carriage among immunized children that has lowered overall rates of spread within the community^3^. In the U.S., these “herd immune” effects of PCV have been particularly impactful for pneumonia in the elderly^4^. Protection by PCV, however, is incomplete, as currently licensed formulations contain only 10 to 20 of the >98 pneumococcal capsular polysaccharide (CPS) serotypes. The 2013 recommendation of PCV for adults has not further decreased the burden of Spn pneumonia, which is more likely to be due to non-PCV serotypes. It has proven immunologically challenging to further increase the number of included CPS serotypes in PCV. Other issues are the high cost of this complex vaccine and rising rates of non-vaccine serotypes (“serotype replacement”) in both carriage and, in studies in some locations, disease^5,6^. Several phase I and II clinical trials of protein-based or whole-cell vaccines have failed to progress, emphasizing the need for new approaches. Since it is estimated that >80% of the public health benefit of PCV is attributable to herd immunity^7^, for any novel strategy to be as effective as PCV its effects would also need to be amplified by disrupting transmission within the population. The only demonstrated means of interrupting pneumococcal community transmission is through a reduction in carriage. While protein-based products have been licensed for another encapsulated pathogen, *Neisseria meningitidis*, it is still unclear how effectively this vaccine protects against carriage^8^. Additionally, because of the widespread use and effectiveness of PCV, a reduction in carriage is likely the only practical, tractable outcome for clinical efficacy studies leading to licensure of novel Spn vaccines^9^.

Spn carriage in the upper respiratory tract (URT), the first step in pneumococcal disease^9^, is characterized by sequential and overlapping episodes, each lasting for weeks to months. Spn carriage rates decline after early childhood for both common and uncommon serotypes^10^. This broad, serotype-independent decline in prevalence with host age is best explained by the gradual accumulation of immunity to conserved Spn antigens. Previous research, using murine models of URT colonization, provide evidence of the development of serotype-independent protection following pneumococcal colonization^11–14^. In this regard, Spn contains highly conserved structural features (such as teichoic acids) and multiple enzymes required to build and remodel its cell surface. Under routine growth conditions in vitro or during invasive infection, the thick layer of CPS effectively covers up many of these antigens. It has been suggested, however, that during carriage there is a down-regulation of CPS, which otherwise blocks adherence and interaction of these bacterial factors with the epithelial surface^15^.

Anti-capsular antibody (Ab) is most effective at impacting colonization when present the time of acquisition, perhaps to promote mucocilliary clearance before the organism can access the epithelial surface or because of the small numbers of incoming bacteria at this time^16^. Targeting genes required during colonization would minimize the possibility that these genes could be lost or mutate in response to vaccine-induced immune pressure. A further consideration is that Spn, like other successful mucosal pathogens of the URT, expresses a protease that cleaves human IgA1, the most abundant immunoglobulin in this niche^17^. Thus, the generation of protease-insensitive IgG might be critical for blocking the establishment of carriage. PCV induces high levels of IgG that access the mucosal surface of the URT by transcytosis^18,19^. Studies in mice and experimental human colonization experiments demonstrate that mucosal IgG to CPS blocks carriage acquisition through its agglutinating function rather than through opsonization^16,20^. This observation established different mechanisms for Ab-mediated protection from carriage and invasive disease^21,22^. Another consideration is that antibody to Spn surface enzymes could interfere with their function and thereby inhibit bacterial growth and viability.

Given these competing immunological factors in both the host and pathogen, empirical studies of the effect of protein vaccine targets in disease models are needed to inform future iterations of the pneumococcal vaccine. We, therefore, tested the hypothesis that specific antibody raised to conserved, immunogenic surface proteins could block Spn colonization. We took an unbiased, whole genomic approach to screen for genes required for URT colonization, as a first step in identification of candidate antigens, which we then validated in our murine infection model.

## Results and discussion

### TnSeq screen to identify loci contributing to colonization

**Fig. 1:**
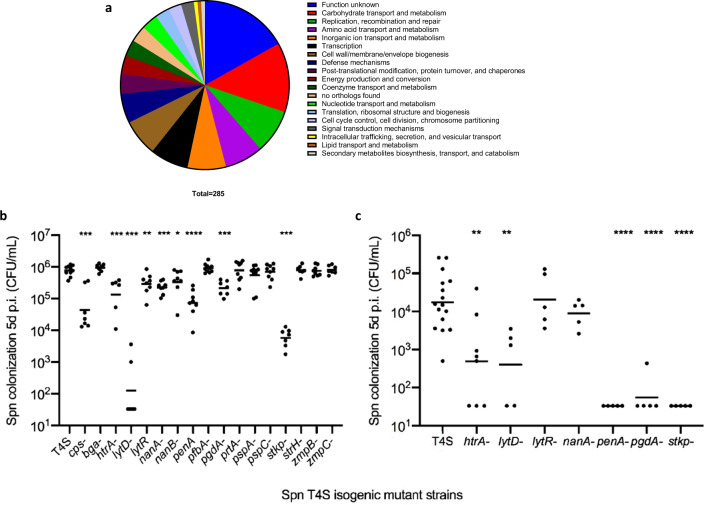
Colonization by TnSeq mutants and analysis of candidate genes. **a** Functional annotation of genes identified in the TnSeq screen with diminished infant mouse colonization (*P* < 0.05). **b** Defined mutants in the 16 genes identified in the TnSeq screen expressing antibody-binding targets were constructed and tested for their ability to colonize infant mice in comparison to the parental (T4S) and unencapsulated (T4*cps*-) strain controls. **c** Mutants with diminished colonization of infant mice (*P* < 0.01) were also tested in adult mice. Symbols represent colonization density (CFU/ml upper respiratory track lavage) of individual mice at 5 days post-infection with median values shown. Statistical comparisons using the Mann–Whitney test for each mutant were to T4S strain. *P < 0.05, **P < 0.01, ***P < 0.001, ****P < 0.0001.

To assess genes impacting Spn colonization using a TnSeq screen, we employed an infant mouse model of pneumococcal colonization^23^, thereby reducing the problem of population bottlenecks (stochastic loss of clones) which confounds whole-genome mutagenesis studies^24^. In contrast to adults, infant mice become stably colonized following a much lower inoculum (50% infecting dose <5 CFU)^25^. Camilli et al. described the use of TnSeq technology in Spn, and we previously described the use of this method in an analysis of genes affecting Spn shedding in our infant mouse model^26–28^. In the current report, we include the analysis of this dataset for genes affecting colonization. Briefly, *Mariner* inserts in TA sites in genomic DNA were generated in vitro and transformed into Spn T4S (a streptomycin-resistant derivative of the extensively-annotated type 4 clinical isolate TIGR4). Twenty-eight independently-generated input pools, containing ~500 unique insertions, were each inoculated into two (biological replicates) 4-day-old pups; URT lavage samples were collected 5 days post-inoculation. The 28 “input” pools were then compared with the corresponding “output” pool. Genomic DNA was isolated from each pool and used to amplify the transposon and insertion site for Ilumina sequencing followed by bioinformatic analysis for statistically significant loss of clones in the output pool. In total, the input pool included >16,800 mutations in non-essential genes (2020 of 2248 TIGR4 open reading frames). We identified a significant loss of fitness for colonization (*P* value cutoff of <0.02) in 198 non-essential genes (TnSeq “hits”; Table S1). [Note: n*anA* (*P* = 0.028) was included in the list because of its potentially overlapping function with *nanB*, making our list 199 candidates^29^]. These “hits” included genes across multiple functional classes (Fig. 1a and Table S1 shown for *P* < 0.05, *n* = 285).

### Prioritization and confirmation of candidate loci

Next, we prioritized immunogenic targets by cross-referencing to a list of 208 binding targets identified from a proteome microarray^30^. These Ab-binding targets (ABTs), which were identified using naturally-acquired serum IgG, were enriched for cell-surface association. No essential gene products were found among the ABTs. Sixteen of our 199 TnSeq ‘hits’ were in ABTs (Table 1). We then constructed defined, knock-out mutants of these candidate genes in T4S and tested these strains for their ability to colonize infant mice (Fig. 1b). Colonization density at 5 days post-inoculation was compared to the T4S parent strain and with a previously described capsule-deficient mutant (T4Δ*cps*) included as a positive control for colonization deficiency^31^. Seven of the 16 candidates showed a significant defect in colonization of infant mice compared to the parent strain (*P* < 0.01). We then assessed colonization of the validated candidates in adult mice as, optimally, a novel Spn vaccine should protect both infants and adults. Colonization of adult mice occurs at a lower density and with increased animal-to-animal variation (Fig. 1c). Of the seven mutant strains attenuated for infant colonization (*P* < 0.01), five were also impaired in colonization of adults (*P* < 0.01). Interestingly, the remaining candidates did not include several well-recognized Spn virulence determinants (PspA, PspC/CpbA, NanA) identified in the TnSeq screen.

**Table 1:** Antibody-binding targets among the TnSeq-identified genes affecting carriage.

Gene ID	Gene	*P*-value	Function
SP_0071	*zmpC*	4.66E−06	Zinc metalloprotease
SP_1732	*stkp*	1.18E−05	Serine/Threonine protein kinase
SP_0930	*cbpE*	0.0001	Choline binding protein E, Also LytD
SP_1833	*pfbA*	0.0001	Plasmin and fibronectin-binding protein A; cell wall surface anchor family protein
SP_0648	*bgaA*	0.0001	Beta-galactosidase
SP_1942	*lytR*	0.0002	Putative transcriptional regulator
SP_1673	*penA*	0.0046	Penicillin-binding protein; pbp2B
SP_0664	*zmpB*	0.0053	Zinc metalloprotease
SP_0057	*strH*	0.0065	Beta-N-acetylhexosaminidase, exoglycosidase
SP_0117	*pspA*	0.0067	Pneumococcal surface protein A
SP_2239	*htrA*	0.007	Serine protease
SP_2190	*pspC/cbpA*	0.0077	Choline binding protein A
SP_1479	*pgdA*	0.0086	Peptidoglycan N-acetylglucosamine deacetylase A
SP_1687	*nanB*	0.011	Sialidase; neuraminidase B
SP_0641	*prtA*	0.0122	Serine protease, subtilase family
SP_1693	*nanA*	0.028	Sialidase; neuraminidase A

**Fig. 2:**
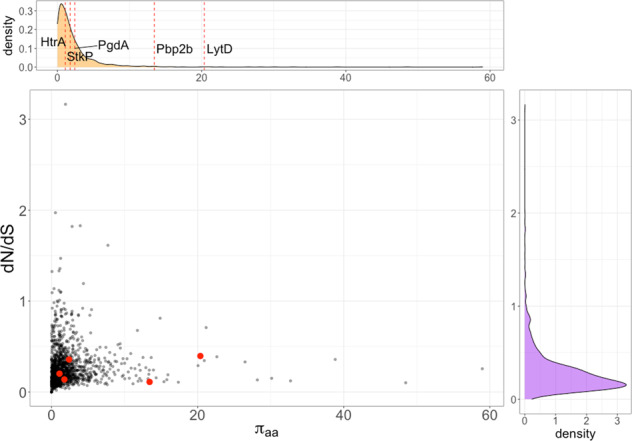
Selection and diversity in core pneumococcal genes and the selected antigens. Top panel: marginal density distribution of π_aa_ (a measure of the average number of coding changes between a pair of sequences from different isolates) showing the position of the selected antigens with red dashed lines, as labeled. Right panel: marginal density distribution of dN/dS (ratio of non-synonymous to synonymous changes). Central panel: scatter plot of average pairwise sequence diversity π_aa_ vs selection strength for all core genes (black) and tested antigens (red).

Each of the remaining five candidate genes are part of the Spn core genome (which consists of 1194 genes found throughout the species)^32^. For each core gene, we produced multiple sequence alignments and quantified selection by calculating the ratio of non-synonymous to synonymous changes (dN/dS) using a maximum-likelihood method (Fig. 2). All of the selected antigens were under negative selection, typical of other functionally conserved bacterial core genes. This suggests rapid evolution away from protein-targeted vaccines via point mutations would be unlikely in any target. We also quantified allelic diversity in the population by calculating π between amino acids^33^, which can be interpreted as the average number of coding changes between a pair of sequences. When a gene has multiple common alleles, π_aa_ will be larger due to their sequence differences (though dN/dS may still be low, as the function of either allele is still conserved). In this analysis, Pbp2b and LytD showed above-average population wide diversity. Pbp2b is a recombination hotspot due to its involvement in penicillin resistance, leading to multiple mosaic alleles^34^. Thus, all candidate immunogenic proteins required for efficient colonization are highly conserved among Spn isolates with PenA and LytD displaying multiple functional conserved alleles.

### Description of protein candidates

All five Spn protein candidates have well-characterized functions on the bacterial cell surface as follows:

StkP is a Ser/Thr protein kinase that functions in the cell division process as a global cell cycle regulator^35^. Four extracellular PASTA domains interact with peptidoglycan fragments at the division septum and activate penicillin-binding protein 2a through StkP-mediated phosphorylation of MacP^36,37^. StkP was also identified in a prior screen of the Spn proteome for increased reactivity with patient convalescent sera following invasive pneumococcal infection^38^. Specific Ab to recombinantly-expressed StkP showed surface staining of encapsulated TIGR4 by FACS analysis. Immunization of mice with the C-terminal portion of StkP (the 314 extracellular AA residues in alum adjuvant) was as effective as PCV in protecting mice against development of Spn pneumonia. However, following a phase I clinical study, because there was no increased killing in an opsonophagocytic assay with sera from StkP immunized individuals, further testing of this antigen was discontinued without consideration of other mechanisms of Ab-mediated protection. In a recent longitudinal study of 976 mother-infant pairs, higher levels of cord blood IgG to StkP were associated with reduced Spn acquisition among infants^39^.

PenA is a monofunctional Class B penicillin-binding protein (Pbp2b) with transpeptidase activity needed for normal cell elongation. AA substitutions in PenA extracellular catalytic domains are associated with low level β-lactam resistance due to decreased drug binding affinity^40^. The selection for β-lactam resistance could explain the higher level of sequence variation for this candidate. A recent report showed that immunization with recombinant PenA conferred significant protection against lethal challenge from diverse Spn strains following intranasal (IN) challenge^41^.

PgdA is an *N*-acetylglucosamine deacetylase that modifies the pneumococcal peptidoglycan backbone^42^. Deacetylation of >80% of GlcNAc residues by PgdA renders the organism resistant to the hydrolytic activity of host lysozyme, an abundant antimicrobial on the mucosal surface of the URT. We have shown that without *pgdA*, Spn grows normally in vitro but is unfit for colonization in a lysozyme-dependent manner^43^. The relatively greater contribution of *pgdA* to colonization in adult compared to infant mice (Fig. 1) correlates with higher expression of lysozyme in adult mice^44^.

HtrA is a serine protease that plays a crucial role in bacterial growth under stress conditions and protein quality control on the bacterial surface^45,46^. For instance, HtrA controls competence and bacteriocin activity by regulating levels of quorum-sensing peptide pheromones that control these functions^47^.

LytD, also known as CbpE or Pce, is a phosphorylcholine esterase that binds to and hydrolyses phosphorylcholine residues on wall- and lipo-teichoic acid protruding from the cell surface^48^. Our group has shown that this enzyme also cleaves and inactivates host platelet activating factor (PAF), a phosphorylcholine-containing substrate^49^. PAF is a potent activator of neutrophils and its depletion by LytD impairs their ability to clear Spn.

### Immunization of adult mice with candidate proteins

The five protein candidates (StkP, PenA, PgdA, HtrA, and LytD) were then tested for their ability to induce immunity that protects against respiratory tract infection. Briefly, the full-length gene (except as noted below) without the signal peptide (based on the TIGR4 reference genome sequence)^50^ was cloned and expressed with N- or C-terminal His tags. StkP contains transmembrane domains and only the C-terminal extracellular domain (AA 345–659) was expressed. Once the purity of recombinant protein candidates was confirmed, these were used to subcutaneously immunize adult mice, with mice receiving one candidate protein plus TiterMax Gold^©^ adjuvant. Our initial focus was on adult mice because we could not induce adaptive immunity directly while mice were still infants. In pilot experiments, we found high titers of serum IgG to the recombinant protein by ELISA, but did not see a significant difference in colonization density compared to adjuvant-only controls following IN challenge with strain T4S. However, colonization levels of both antigen+adjuvant and adjuvant-alone groups were lower than naïve age-matched control mice, suggesting a confounding antigen-independent effect of immunization (Supplementary Fig. 1A). Prolonged effects of adjuvants on respiratory tract infection by Spn has been reported previously^51^.

**Fig. 3:**
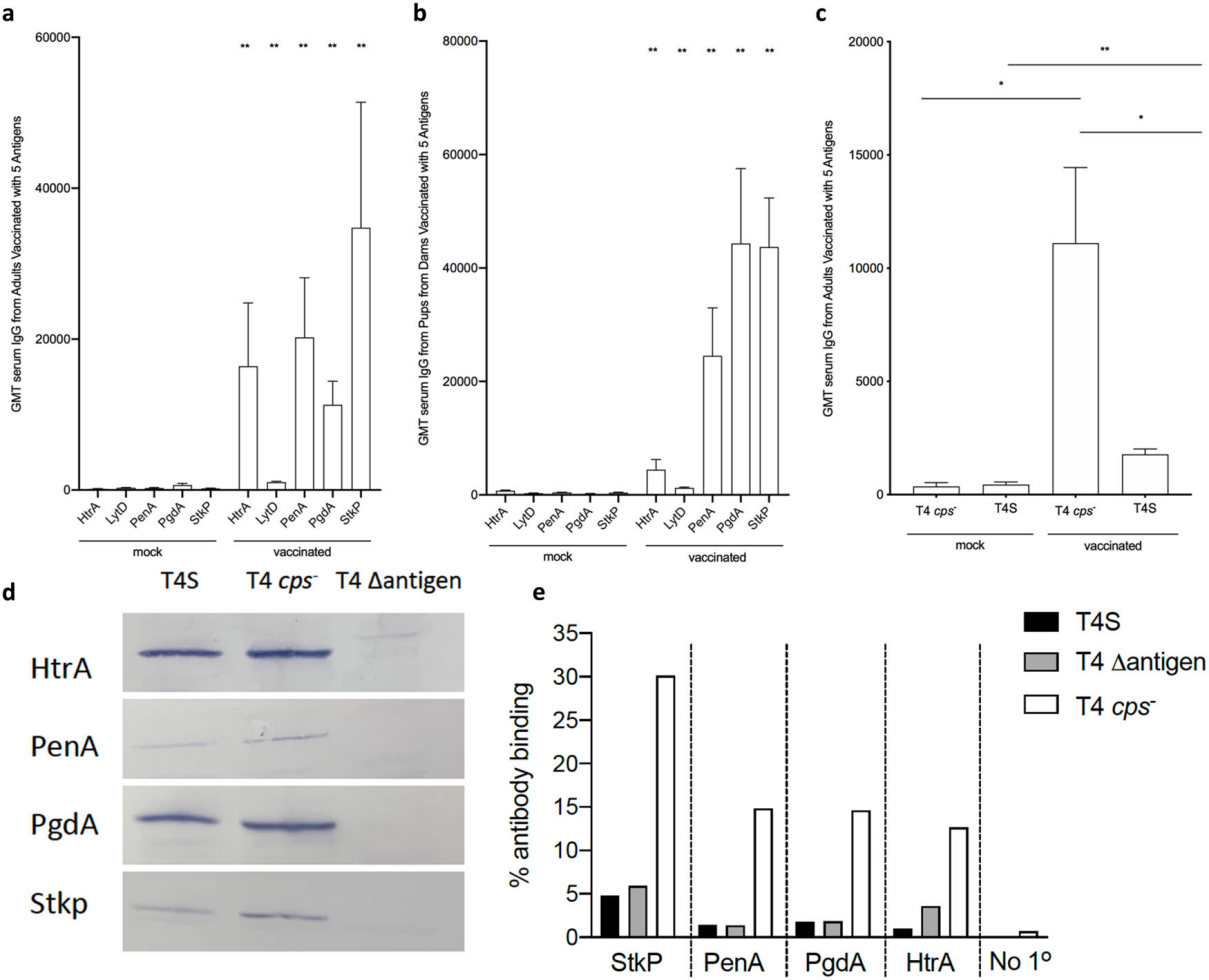
Effect of immunization on antibody responses. Dams were vaccinated with the five-antigen combination (StkP, PenA, PgdA, HtrA, and LytD) with adjuvant and compared to adjuvant alone controls (mock). Serum from **a** adults (*n* = 6) and **b** pups from vaccinated dams (*n* = 5) was used to quantify serum IgG responses to individual antigens by ELISA. Statistical significance of ELISA values was determined using a Mann–Whitney test. **c** Adult sera were also used in a whole bacterial cell ELISA using the unencapsulated mutant (T4*cps–*) or its encapsulated parent strain (T4S). Values represent geometric mean titers ± S.E.M. ELISA values were compared using an unpaired Student’s *t* test. *P < 0.05, **P < 0.01. **d** *P*ooled sera (*n* = 5) from adult mice immunized with the individual antigen shown was used in Western analysis on whole-cell lysates of strain T4S, the unencapsulated mutant T4*cps−* or the mutant construct corresponding to the sera tested. **e** Representative flow cytometric comparison of antibody binding to the surface of strain T4S, the unencapsulated mutant T4*cps*−) or the mutant construct corresponding to the sera tested. Samples tested were pooled sera (*n* = 5) from adult mice immunized with the individual antigen shown below or a no sera control.

To further optimize protection, we immunized adult mice with a combination of all five candidate proteins + TiterMax Gold^©^ adjuvant. This resulted in high titers of serum IgG to each of the candidate proteins as measured by ELISA, although the response to LytD was less robust compared to the other four antigens (Fig. 3a). Importantly, a whole bacterial cell ELISA to strain T4S was used to demonstrate that the levels of serum IgG following immunization increased antibody recognition of the Spn surface (Fig. 3c). Whole bacterial cell ELISA with serum from the adults immunized with individual proteins showed that antibody to no single protein accounted for this surface recognition. Western analyses of whole-cell lysates confirmed that immunization generated specific antibody for each of the four antigens against which antibody was generated at high titer (Fig. 3d). Total IgG obtained from immune serum (at a concentration as high as 800 μg/mL) did not show agglutinating activity of either strain T4S or T4Δ*cps*. Moreover, immune serum showed no inhibitory effect on in vitro growth of strain T4S in nutrient medium (Supplementary Fig. 1B). The lack of an effect on growth was not unexpected as only the T4Δ*stkp* mutant demonstrated impaired growth characteristics.

### Protection from pneumococcal colonization

**Fig. 4:**
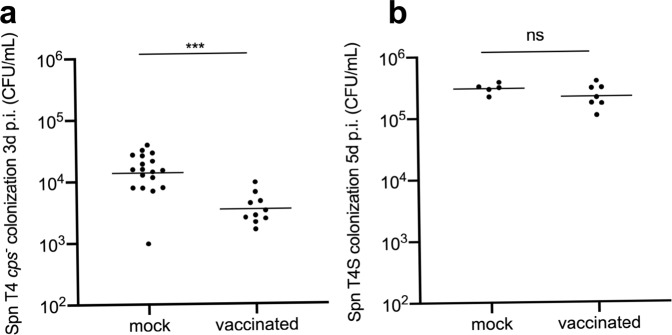
Effect of immunization on infant mouse colonization. Dams were vaccinated with the five-antigen combination (StkP, PenA, PgdA, HtrA, and LytD) with adjuvant and compared to adjuvant alone controls (mock). After the immune response was confirmed, they were bred and pups were challenged with the **a** unencapsulated mutant (T4*cps-*) or **b** its encapsulated parent strain (T4S). Symbols represent colonization density (CFU/ml upper respiratory track lavage) for individual mice at 3 (T4*cps*-) or 5 (T4S) days post-infection with median values shown. Mann–Whitney test used for comparisons. ***P < 0.001, n.s., non-significant.

The confounding effect of the adjuvant on colonization was also observed when adult mice immunized with the five-antigen combination were IN challenged (Fig. S1A). Due to the difficulties in assessing pneumococcal colonization in immunized and adjuvant-only adult mice, we examined protection from colonization in infant mice, who acquire maternal antibody in utero and post-natum^52^. Serum IgG levels to each of the five recombinant proteins were comparable between infants from vaccinated dams and adults immunized with the combination of antigens (Fig. 3b). Four-day-old pups from immunized dams and pups from adjuvant-only dams were challenged IN with a low dose (10^3^ CFU) of strain T4S. Expected levels of colonization at 5 days post-inoculation were achieved in adjuvant-only pups, but there was no protection in the vaccinated pups (Fig. 4) despite high levels of serum IgG. We also tested the effect of immunization on protection among pups using a model of intra-litter transmission in the setting of influenza A co-infection where on average <10 bacteria establish infection^25^. Transmission of strain T4S from inoculated index pups was observed in 54.5% of contact pups in litters from immunized dams compared to 78.6% in litters from dams given adjuvant alone (*P* = 0.175, Table S2). This result contrasted with the complete protection previously observed in pups from dams immunized with PCV^52^. The lack of robust protection with the combination of five candidate antigens led us to question whether the pneumococcal capsule might be interfering with antibody binding to its targets in vivo. To examine this possibility, we repeated the whole bacterial cell ELISA with an unencapsulated mutant of strain T4S (T4Δ*cps*). Using the same pool of sera as in Fig. 3a, we found that surface binding of IgG from vaccinated mice was far greater to the unencapsulated strain compared to the isogenic encapsulated parent strain (Fig. 3c). Similarly, a comparison of antibody binding using flow cytometry on pneumococci showed that immune, high titer sera generated to the individual proteins recognized the bacterial surface but only when not expressing capsule (Fig. 3e). Unlike challenge with T4S, when pups received 10^3^ CFU T4Δ*cps*, immunity to the combination of five Spn proteins was associated with decreased colonization density compared to adjuvant-only pups (Fig. 4). [Note: colonization density was assessed at 3 rather than 5 days p.i. because of its shorter duration for unencapsulated strains^53^]. Western analysis confirmed that protection in immunized infant mice against strain T4Δ*cps* colonization was not due to increased antigen expression in the absence of capsule (Fig. 3d).

### Summary

Our unbiased genome-wide approach using Tn-Seq identified multiple conserved, immunogenic, surface exposed proteins required for Spn colonization. After identifying and validating these targets, we tested whether antibody-mediated disruption of their function could mitigate pneumococcal carriage. We demonstrated that, in mice, antibodies generated by systemic immunization with a combination of five recombinant pneumococcal surface proteins was sufficient to reduce Spn colonization in the nasopharynx. However, this protective effect was observed only when the pneumococcal capsule was not present—a result that correlated with increased binding of serum antibody to the surface unencapsulated pneumococci^54^. Protection against the uncapsulated strain in infant mice, who develop immunity through passive transfer, makes a role for T cell rather than antibody-mediated protection unlikely. Of course, we cannot exclude the possibility that the antibody generated by immunization with these targets could be protective against encapsulated Spn if induced at higher titers. It is also possible that our approach, while not sufficient to block colonization, could be effective at later stages of pathogenesis or that antibody directed against selected other conserved, immunogenic surface targets alone or in combination could provide protection against encapsulated Spn. In this regard, leading protein vaccine candidates, including PspA and PspC(CbpA), were identified in our TnSeq screen. As these have variable domains, it appears they are under immune pressure, perhaps because they extend beyond the capsule unlike the five candidates we tested. Mutants in *pspA* and *pspC*, however, showed wild-type levels colonization and, therefore, were not considered further. It also remains plausible that a combination of capsular polysaccharide and common protein antigens could be used to optimize and broaden protection^55,56^. That said, our study provides a demonstration of the challenge of serotype-independent approaches to prevent Spn infection: the effectiveness of the capsule in shielding conversed surface antigens that would otherwise be targets of protective humoral immunity.

## Methods

### Bacterial strains and culture

*S. pneumoniae* strains were grown in tryptic soy (TS) broth (BD, Franklin Lakes, NJ) at 37 °C, without aeration, to an optical density at 620 nm (OD_620_) of 1.0; or incubated on TS agar plates supplemented with 100 μl of catalase (30,000 U/ml; Worthington Biochemical) and antibiotic (as indicated) at 37 °C in 5% CO_2_, overnight. Antibiotics used in this study are as follows: streptomycin (str; 200 μg/ml); kanamycin (kan; 125 µg /mL or 500 µg/mL); erythromycin (erm; 1 or 2 µg/mL); spectinomycin (spec; 200 µg/l); and chloramphenicol (cam; 2 µg/ml).

### Mutant construction and protein purification

The SpnΔ*lytR* SpnΔ*nanB*, SpnΔ*penA*, SpnΔ*pfbA*, and SpnΔ*stkP* knockout strains were constructed in a str-resistant *S. pneumoniae* type T4 strain (T4S, P2406) using the Janus cassette^57^. The Janus cassette was amplified from genomic DNA, obtained using a MasterPure DNA purification kit (Illumina), from Spn T4 strain P2408^58^, with upstream and downstream regions (~1 kb) flanking to the gene of interest added via isothermal assembly. Strain P2406 was then transformed with the PCR product, and the transformants were selected on 5% blood agar plates supplemented with kan (500 µg/mL). The gene replacements with the Janus cassette were confirmed by PCR. The SpnΔ*zmpB* and SpnΔ*zmpC* Janus-insertions strains were kindly provided by Dr. Pyong Woo Park^59^, and the deletions were moved to our T4S parent strain by transforming genomic DNA obtained from them into P2406, and selecting the transformants on TS-kan (500 µg /mL) plates and 5% sheep’s blood agar plates. Strains SpnΔ*pgdA* (P1831; kan^R^), SpnΔ*pspA* (P2205; kan^R^), SpnΔ*bga* (P2167; erm^R^), SpnΔ*htrA* (P1539; kan^R^), SpnΔ*strH* (P2168; spec^R^), SpnΔ*nanA* (P2082; cam^R^) and SpnΔ*pce/lytD* (P2421; kan^R^), were constructed by transforming genomic DNA obtained from them into P2406, and the transformants selected on TS-agar plates with appropriate antibiotics. The cbpA/pspC deletion was constructed by transforming genomic DNA obtained from P1465 in to P2406 and selecting on TS-agar plates supplemented with erythromycin (2 µg/ml) and 5% blood. DNA obtained from this strain was back-transformed in to P2406 and transformants were selected on plates as described above, to generate strain P2513 (*cbpA::erm*; Erm^R^, Strep^R^).

For purification of recombinant proteins, the pET-28a(+) vector (Novagen, Madison, WI), which contains a his_6_ tag at the N-terminus, was used. The open reading frames for genes *htrA*, *penA*, *pgdA*, *pce/lytD*, and *stkp* were amplified by PCR using Q5 polymerase (NEB) with genomic DNA as template and primers that contained restriction enzyme sites for NdeI and XhoI (*htrA*, *pgdA*, *stkP*, and *penA*) and NheI and XhoI (pce/*lytD*), and were cloned in to the pET-28a(+) vector. The resulting plasmids were transformed into a derivative of *E. coli* strain BL21(DE3)pLysS that had been modified to have a deletion of *ompA* (making strain E548). DegP has homology to HtrA and has been shown to co-purify with rHtrA^46^; therefore, we deleted the *E. coli* gene coding for the protease DegP by P1*vir* phage transduction of DNA from strain (E936) with *degP::kan* from Keio collection^60^ followed by excision of the kanamycin-resistance cassette by expression of the FLP recombinase from temperature sensitive plasmid pcp20 (E947). For rPce, the first 26 amino acids of *pce/lytD* were not included, as they likely contain the signal sequence. As described previously^38^, only the C-terminal immunogenic PASTA domain region of StkP (AA 345–659) was cloned in to the expression vector.

Recombinant proteins were purified with the HisTrap column (GE Healthcare) by FPLC using Äkta Start (GE Healthcare)^35,38,46,61–63^. Briefly, *E. coli* cell pellets were resuspended in binding buffer (20 mM NaH_2_PO_4_, 500 mM NaCl, 20 mM Imidazole, 5% glycerol, 0.5% Triton, 10 mM 2-mercaptoethanol pH 7.4 and 20 mg/ml lysozyme), loaded on the HisTrap column, washed to remove non-specific bound protein (10-15% of elution buffer mixed with binding buffer), and fractions were eluted in 1 ml (elution buffer is binding buffer with the addition of 500 mM imidazole). For rStkP C-terminal purification, the cell pellet was first resuspended in binding buffer with 20 µl of RNAse A (0.5 mg/ml) and 20 µl of DNAse I (150 units/ml), incubated on ice for 60 min, and vortexed every 10 min. Samples were then sonicated (10 times for 30 s at 10% output, with 30 s on ice in between each sonication step) and pelleted by centrifugation (10,000 × *g* for 30 min at 4 ^o^C). The pellet, containing the insoluble fraction, was solubilized in 8 M urea, 50 mM Tris-Cl, pH 8, sonicated (as described above) and pelleted by centrifugation (15,000 *g* × 10 min, RT). The supernatant was loaded on to the HisTrap column, washed, and eluted as described above. Fractions containing the proteins were pooled together and dialyzed with the following dialysis buffer: rPenA (25 mM Tris-Cl pH 8.0, 5% glycerol, 100 mM NaCl); rPce/LytD (25 mM Tris-Cl pH 8.0, 5% glycerol, 100 mM NaCl); rStkP-C terminal (25 mM Tris-Cl pH 7.4, 10% glycerol, 100 mM NaCl); rHtrA (PBS); and rPgdA (50 mM Na_2_HPO_4_, 150 mM NaCl, and 0.5 mM CoCl_2_ pH 7.5). Recombinant proteins were concentrated using Amicon Ultra centrifuge filters (Millipore; according to the manufacturer’s instructions). Protein concentration was assessed by Bradford protein assay (Bio-Rad cat. no. 5000006; bovine serum albumin Protein Standard II cat. no. 5000007).

### Mouse studies

Wild-type C57BL/6J mice (Jackson Laboratories, Bar Harbor, ME) were used in all experiments. Mice were maintained and bred in a conventional animal facility. Pups were housed with a dam for the course of the studies. Following infection, all mice appeared healthy, showed normal activity, and gained weight similar to uninfected controls. These studies were conducted in accordance with the recommendations of the Guide for the Care and Use of Laboratory Animals. All mouse studies were approved by the Institutional Animal Care and Use Committee of the New York University Medical Center. All procedures were in compliance with Biosafety in Microbiological and Biomedical Laboratories.

### Transposon library preparation and in vivo selection

Library construction using the mariner transposon (Tn) was carried out as previously described; we generated 28 independent libraries^28^. Each library contained ~500 random transposon insertion mutants in nonessential genes. Pools were stored at −80 in 20% glycerol-TS until use. For mouse infections, a transposon pool was grown in TS broth in static culture at 37 °C. Two 4-day-old pups (representing biological replicates) were inoculated IN, without anesthesia, with 4000–8000 CFU Spn in 3 μL PBS, such that each pup received ~10-fold excess of the Tn mutant strains (*Input*).

On day 5 post-inoculation, pups were euthanized by CO_2_ and cardiac puncture; retrotracheal lavages were collected from the nares (200 µL PBS). Lavages were diluted and plated to TS-str plates supplemented with catalase and incubated at 37 °C + 5% CO_2_ to determine colonization density. The remainder of the lavage was plated on TS-str plates supplemented with catalase and incubated overnight at 37 °C + 5% CO_2_. After overnight incubation, the colonies were collected from the plate and transferred to 5 mL sterile PBS (Output). Genomic DNA was isolated and samples were prepared as described^28^. A clean up step was added to the ligation mixture to remove excess adaptor using AMPure XP beads (Beckman Coulter) before using PCR to amplify the transposon insertion site.

### Sequencing and TnSeq bioinformatics analysis

Transposon-junction DNA fragments were subjected to single-end 50 rapid run sequencing on an Illumina HiSeq2500 Instrument (Illumina, San Diego). The resulting reads were filtered, mapped, and normalized as described^64^. Reads were aligned using Bowtie^65^ to the *S. pneumoniae* TIGR4 reference genome^50^ and insertion sites were called for each sample based on the alignment counts. Insertion sites within 80% of gene length from the transcription start sites were treated as candidates that could affect gene functions. Finally, TnseqDiff^66^ was used to compare the insertions between output (nasal lavage) and input (inoculum) samples to identify candidate genes required for URT colonization. Functional/biological annotation was carried out using EggNOG(v5)^67^.

### Sequence conservation of candidate antigens

To quantify the conservation and selection pressure on the chosen antigens, we used the publicly available sequences from the same study in which their IgG binding was measured^30^. This consisted of 616 *S. pneumoniae* samples isolated from the nasopharynx of asymptomatic child carriers, over seven years spanning the rollout of PCV-7^68^. We used a previously-generated core-genome alignment, with clusters of orthologous genes (COG) as defined within this^69^. We then calculated dN/dS values for each COG as follows: we first aligned each COG at the codon level using RevTrans^70^ and at the amino acid level using MUSCLE^71^. We used the nucleotide alignment to generate a maximum likelihood tree using a GTR rate model with IQ-TREE^72^. These alignments and the tree were then input into HYPHY, where we used Single-Likelihood Ancestor Counting^73^ to calculate dN/dS values for each COG. We calculated the pairwise-diversity of the translated sequences (p_aa_) using dendropy on the amino-acid alignments^74^.

### Vaccination and spn challenge of adult mice

Four-week-old male and female mice were vaccinated with 5ug of each recombinant protein + an equal volume of TiterMax Gold^©^ adjuvant or a combination of all 5 proteins (5 µg each; 25 µg total) + TiterMax Gold adjuvant; mock mice received an equal amount of adjuvant only. The injection was divided and mice were vaccinated by subcutaneous injection in 2–3 different body sites (back of the neck, base of tail, and flank), two times at an interval of 14 days. Mice were challenged with Spn 8-weeks post-vaccination (6-weeks post-boost). Briefly, mice were infected intranasally (IN) with 10^5^ CFU Spn T4S in 10 μL of sterile phosphate-buffered saline (PBS) by instillation on the nares with a blunt pipette tip, without anesthesia. On day 5 post-inoculation, mice were euthanized by CO_2_ and cardiac puncture; retrotracheal lavages were collected from the nares (in 200 µL PBS). Lavages were diluted and plated to TS-str plates supplemented with catalase and incubated at 37 °C + 5% CO_2_ to determine colonization density. The presence of specific IgG in the serum of mice was confirmed by ELISA (as described below).

### Maternal immunization and Spn challenge of pups

Immunity to the recombinant pneumococcal proteins was induced in adult female mice as described above. The female mice were then paired with males, and their litters were used in experiments to assess the role of immunity in protecting pups from pneumococcal challenge. Four-day-old pups were infected with 10^3^ CFU of Spn T4S or T4Δcps in 3 μL of PBS by IN instillation with a blunt pipette tip, without anesthesia, and returned to their mother for the duration of the experiment. Three- or five-days post-inoculation, pups were euthanized by CO_2_ asphyxiation and cardiac puncture and the trachea was lavaged with 200 μL of sterile PBS, which was collected from the nose. Tenfold serial dilutions of the lavage sample were plated to TS-str plates and enumerated after overnight incubation. The presence of anti-pneumococcal IgG in serum of mothers and their pups was confirmed by an ELISA (as described below). Transmission of Spn T4S among pups from vaccinated (5 antigen+adjuvant) compared to mock dams (adjuvant alone) in the setting of influenza A (strain x31) co-infection was examined using a previously described protocol with a ratio of ~1 to 4 index:contact pups^52^.

### ELISA

An ELISA to assess the titer of anti-pneumococcal IgG in the serum of immunized mothers and their pups, using whole bacteria as the capture antigen, was done as previously described^11^. Briefly, Immulon 2HB flat-bottom microtiter plates were coated with 100 μl of PBS-washed whole bacteria (*S. pneumoniae* T4S or T4Δcps), diluted in coating buffer (0.015 M Na_2_CO_3_, 0.035 M NaHCO_3_) to a final OD_620_ of 0.1, and incubated at 4 °C overnight. The plates were then washed three times with wash solution (0.05% Brij-35 Surfact-Amps [Thermo, Fisher Scientific]–PBS) and blocked for 1 h at room temperature with 1% bovine serum albumin (BSA)–PBS. Serial serum dilutions, in PBS, were applied to the plates and incubated at 4 °C overnight. The plates were then washed three times with wash solution. Mouse IgG was detected with alkaline phosphatase (AP)-conjugated goat anti-mouse IgG antibody (Sigma A5153) at a 1:4,000 dilution in PBS, incubated at 37 °C for 1.5 h. The plates were then washed three times with wash buffer. The plate was developed with *p*NPP substrate tablets (Kirkegaard & Perry Laboratories Inc., Gaithersburg, MD) dissolved in diethanolamine (DEA [Thermo, Fisher Scientific]). Absorbance at 405 nm was read at 1 h. The endpoint titers were determined by calculating the dilution at which the absorbance is equal to 0.1.

An antigen ELISA assessed the presence of anti-pneumococcal protein IgG in the serum of mice vaccinated with recombinant proteins. Purified recombinant proteins (the same used to vaccinate mice), assessed individually, were diluted to a final concentration of 2 μg/ml in PBS and applied to Nunc-Maxisorp flat-bottom microtiter plates. Plates were incubated overnight at 4 °C and then washed three times with PBS and blocked for 2 h at RT with 1% BSA–PBS. Serially diluted serum samples, in 1% BSA–PBS, were applied to the plate and incubated at 37 °C for 1 h. The plate was washed with PBS, and 100 μl of AP-conjugated goat anti-mouse IgG (diluted 1:2,500 in PBS) was applied to each well. The plate was incubated at 37 °C for 1 h. The wells were washed with PBS, and the plate was developed as described above.

### Western blot analysis

Bacteria were grown to an OD_620_ = 0.8, centrifuged, washed in PBS. After resuspension of the pellet in NuPAGE LDS sample buffer with NuPAGE reducing agent (Invitrogen), lysates were prepared by treatment at 100 °C for 5 min and storage at −20 °C. Lysates were separated using 10% Bolt Bis-Tris Plus gels (Thermo Fisher Scientific NW00100BOX) and transferred onto PVDF membranes via a dry blotting system (iBlot 2, Thermo Fisher Scientific). Membranes were blocked with 1% bovine serum albumin/PBS for one hr. Subsequent steps were carried out in TBS/0.1% Tween-20. Blots were incubated with pooled immune sera (1:500) overnight at 4 °C and then, following 3 washes, with an alkaline phosphatase–coupled goat anti-mouse IgG (1:5000, Sigma A3688) for 2 h at RT. Protein bands were visualized after 3 washes using BCIP/NBT as substrate. For detention of each antigen, all blots derive from the same experiment and were processed in parallel (Supplementary Fig. 2).

### Flow cytometric analysis

Bacterial cultures were grown to an O.D. of 0.2 at 620 nm, resuspended in PBS and incubated with 1% sera from adult mice immunized with each protein candidate at 37 °C for 30 min. Cells were washed, then incubated with an APC-conjugated anti-mouse secondary antibody (Invitrogen) at 1:100 in 2% BSA in PBS at 4 °C for 30 min. Cells were washed, then fixed in 2% paraformaldehyde at room temperature for 20 min. Samples were run on an LSRII flow cytometer (BD Biosciences) and FACS analysis was performed using FlowJo software (Tree Star). Antibody binding was determined by proportion of bacteria with APC fluorescence signal with the gating strategy shown in Supplementary Fig. 3.

### Statistical analysis

Statistical analysis was done using GraphPad Prism 8.0 (GraphPad Software, Inc., San Diego, CA).

### Reporting summary

Further information on research design is available in the Nature Research Reporting Summary linked to this article.

## Supplementary information

Supplementary Information

Reporting Summary

## Data Availability

All datasets generated during and/or analyzed during the current study are available from the corresponding author on reasonable request.

## Code availability

Code used to analyze selection and diversity in the gene sequences is available on github (MIT license) https://github.com/johnlees/pneumo_core and archived at 10.5281/zenodo.5606975. External software used (and version): RevTrans (v.1.10); MUSCLE (v.3.8.31); IQ-TREE (v.1.5.5); HYPHY (v.2.3); dendropy (v4.4.0).
