# Supplementary material for: Pneumococcal capsule blocks protection by immunization with conserved surface proteins
Source: NPJ Vaccines. 2021 Dec 20;6:155. doi: 10.1038/s41541-021-00413-5 (PMC8688510; doi:10.1038/s41541-021-00413-5)
Supplement: Supplementary file 1 — Supplementary Information [file 41541_2021_413_MOESM1_ESM.pdf]

Supplementary Table 1: Genes Identified by TnSeq Screen

	Input vs NL					# Key
Gene Name; TIGR4 Designation	pvalue (Input vs NL)	log2FC (NL/Input)	Total Inserts (Input)	Unique Inserts (Input)	Gene function	Biological Process
SP_0403; rnhC	1.23E-07	3.00E-06	34	24	ribonuclease HIII	Replication, recombination and repair
SP_0873	2.50E-07	2.89E-15	87	61	membrane protein	Cell cycle control, cell division, chromosome partitioning
SP_1207; xseA	5.92E-07	0.00012137	49	37	exodeoxyribonuclease VII, large subunit	Replication, recombination and repair
SP_0186; uvrA	7.11E-07	3.15E-07	100	80	excinuclease ABC, subunit A	Replication, recombination and repair
SP_0369; pbp1A	1.44E-06	6.35E-08	96	69	penicillin-binding protein 1A	Cell wall/membrane/envelope biogenesis
SP_0738	2.29E-06	0.022250721	13	12	hypothetical protein	no orthologs found
SP_0897; pyk	3.00E-06	0.000318477	59	52	pyruvate kinase	Carbohydrate transport and metabolism
SP_1081; murA-1	3.40E-06	2.32E-09	61	39	UDP-N-acetylglucosamine 1-carboxyvinyltransferase	Cell wall/membrane/envelope biogenesis
SP_0071; zmpC	4.66E-06	3.35E-09	434	285	Zinc metalloprotease ZmpC	no orthologs found
SP_1732	1.18E-05	2.11E-06	73	63	serine/threonine protein kinase	Signal transduction mechanisms
SP_0475	1.37E-05	0.000411253	159	105	hypothetical protein	Function unknown
SP_0690; divIB	2.31E-05	1.09E-05	54	42	cell division protein FtsQ	Cell cycle control, cell division, chromosome partitioning
SP_0045	2.37E-05	2.88E-06	149	115	hosphoribosylformylglycinamidine	Nucleotide transport and metabolism
SP_0975	3.43E-05	9.58E-09	92	70	exoribonuclease, VacB/Rnb family	Transcription
SP_0200; ccs4	3.57E-05	4.39E-08	64	45	competence-induced protein Ccs4	Function unknown
SP_0730; spxB	3.64E-05	0.005293476	114	60	pyruvate oxidase	Amino acid transport and metabolism, Coenzyme transport and metabolism
SP_1821	3.67E-05	0.022778893	56	47	sugar-binding transcriptional	Transcription
SP_1445; guaA	4.46E-05	1.46E-06	79	58	GMP synthase	Nucleotide transport and metabolism
SP_1830	5.98E-05	0.006061035	51	38	putative phosphate transport system	Inorganic ion transport and metabolism
SP_0536; blpL	6.26E-05	0.001421674	18	16	immunity protein BlpL	Function unknown
SP_1724; scrB	6.47E-05	9.59E-10	67	48	sucrose-6-phosphate hydrolase	Carbohydrate transport and metabolism
SP_0681	7.14E-05	8.66E-06	68	54	elongation factor Tu family protein	Translation, ribosomal structure and biogenesis
SP_0742	7.45E-05	0.001100517	39	28	conserved hypothetical protein	Function unknown
SP_1890; amiC	8.65E-05	5.87E-05	67	51	oligopeptide ABC transporter,	Inorganic ion transport and metabolism
SP_1087; pcrA	9.69E-05	0.000263212	70	63	ATP-dependent DNA helicase PcrA	Replication, recombination and repair
SP_0737	0.000105976	6.75E-07	66	43	sodium-dependent transporter	Function unknown
SP_0930; cbpE	0.000108646	8.32E-05	105	87	cbpE; choline binding protein E	Function unknown
SP_1833	0.000112528	0.000268353	150	107	protein A | cell wall surface anchor family protein	Inorganic ion transport and metabolism
SP_0648; bgaA	0.000122951	9.64E-06	309	222	bgaA; beta-galactosidase	Carbohydrate transport and metabolism
SP_1119; gapN	0.000149821	0.000116389	58	35	glyceraldehyde-3-phosphate	Energy production and conversion
SP_1942	0.000170729	0.01770152	39	35	putative transcriptional regulator	Transcription
SP_0618; uvrC	0.000182592	7.45E-06	73	56	excinuclease ABC, subunit C	Replication, recombination and repair
SP_1506	0.000192099	3.56E-07	52	33	6-phosphogluconolactonase	Carbohydrate transport and metabolism
SP_0006; mfd	0.000196159	3.44E-07	178	134	transcription-repair coupling factor	Replication, recombination and repair
SP_1229; fhs	0.000216008	0.010388824	48	42	formate--tetrahydrofolate ligase	Coenzyme transport and metabolism
SP_0981	0.000281017	8.31E-05	50	30	foldase protein PrsA, putative protease maturation protein	Post-translational modification, protein turnover, and chaperones
SP_0346; cps4A	0.000303791	0.031602065	59	50	capsular polysaccharide biosynthesis	Transcription
SP_2007; nusG	0.000306515	2.72E-05	32	24	transcription antitermination protein	Transcription
SP_0929; rluD	0.000376515	0.030458287	27	23	ribosomal large subunit pseudouridine synthase D	Translation, ribosomal structure and biogenesis
SP_1723	0.000378631	0.000362038	26	16	hypothetical protein	Function unknown
SP_1895; rafG	0.000490359	0.041604112	45	28	sugar ABC transporter, permease	Inorganic ion transport and metabolism
SP_1075; cpoA	0.000531907	0.000849643	47	42	glycosyl transferase CpoA	Cell wall/membrane/envelope biogenesis
SP_0570	0.000545179	0.01413313	115	98	conserved domain protein	Replication, recombination and repair
SP_1768	0.000562921	5.12E-06	56	40	conserved hypothetical protein	Cell wall/membrane/envelope biogenesis
SP_0013; ftsH	0.000576868	0.000319047	82	70	cell division protease FtsH	Post-translational modification, protein turnover, and chaperones
SP_0358; cap4J	0.000584817	1.05E-06	38	26	capsular polysaccharide biosynthesis protein Cps4J	Carbohydrate transport and metabolism, Cell wall/membrane/envelope biogenesis
SP_0978; coiA	0.000643366	1.69E-07	60	36	competence protein CoiA	Function unknown
SP_2057; adr	0.0006448	0.000325716	97	71	conserved hypothetical protein	Lipid transport and metabolism
SP_1176; ptsI	0.000666475	0.000230252	55	46	phosphoenolpyruvate-protein	Carbohydrate transport and metabolism
SP_0705	0.000699419	2.99E-05	94	62	hypothetical protein	Function unknown
SP_1151; rexB	0.000742892	0.045738202	94	85	ATP-dependent helicase/nuclease subunit B rexB; exonuclease RexB	Replication, recombination and repair
SP_0325	0.000751738	0.002607419	37	27	PTS system, IID component	Carbohydrate transport and metabolism
SP_0303; bglA-1	0.000776677	0.000479694	114	71	6-phospho-beta-glucosidase	Carbohydrate transport and metabolism
SP_1413; hprK	0.000790711	0.003493596	30	23	Hpr(Ser) kinase/phosphatase	Signal transduction mechanisms
SP_1261	0.00080298	0.016522545	32	23	conserved hypothetical protein	Nucleotide transport and metabolism
SP_0549	0.000880833	0.000592763	27	22	conserved hypothetical protein	Cell wall/membrane/envelope biogenesis
SP_2010; pbp2A	0.000899469	1.44E-05	94	77	penicillin-binding protein 2A	Cell wall/membrane/envelope biogenesis
SP_0706	0.000936283	0.000502803	48	37	hypothetical protein	Function unknown
SP_2147	0.000956312	0.000765169	18	13	hypothetical protein	no orthologs found
SP_1982	0.000981483	0.00019287	22	17	thiamine pyrophosphokinase	Coenzyme transport and metabolism
SP_0377; cbpC	0.001006281	2.47E-07	84	47	choline binding protein C	Function unknown
SP_0282	0.001048845	0.000613565	60	39	PTS system, mannose-specific IID	Carbohydrate transport and metabolism
SP_1315; ntpD	0.001118456	0.003318477	53	41	v-type sodium ATP synthase, subunit	Energy production and conversion

SP_1449; cppA	0.001236367	0.001108362	50	34	Catabolite control protein A	no orthologs found
SP_0447; ilvC	0.001288715	0.000224623	59	41	ketol-acid reductoisomerase	Amino acid transport and metabolism, Coenzyme transport and metabolism
SP_1343	0.001292006	4.88E-07	105	79	prolyl oligopeptidase family protein	Amino acid transport and metabolism
SP_0483	0.001444846	0.00084003	74	57	ABC transporter, ATP-binding protein	Inorganic ion transport and metabolism
SP_1635	0.001520903	0.002303237	29	19	hypothetical protein	Function unknown
SP_0042; comA	0.001692061	0.007671732	121	88	competence factor transporting ATP-	Defense mechanisms
SP_0525; blpS	0.001722809	0.036450821	33	20	BlpS protein	Transcription, Signal transduction mechanisms
SP_1883	0.001728493	3.68E-05	71	49	[EC:3.2.1.93] | (GenBank) putative dextran glucosidase DexS	Carbohydrate transport and metabolism
SP_1371; aroA	0.001766202	0.000647972	74	53	3-phosphoshikimate 1-	Amino acid transport and metabolism
SP_0301	0.00188807	6.11E-05	12	9	glycosyl hydrolase, family 1,	Function unknown
SP_1378	0.001959136	0.011534567	49	39	23S rRNA (cytosine1962-C5)-methyltransferase	Translation, ribosomal structure and biogenesis
SP_2159	0.002001722	2.04E-07	278	172	fucolectin-related protein	Function unknown
SP_0284; manL	0.002022292	7.13E-05	26	22	PTS system, mannose-specific IIAB	Carbohydrate transport and metabolism
SP_1129	0.002035546	9.48E-06	58	40	integrase/recombinase, phage	Replication, recombination and repair
SP_0599; vex1	0.002049761	0.004199093	61	46	putative ABC transport system	Defense mechanisms
SP_1057	0.002052426	0.001018128	74	45	putative transcriptional regulator PlcR	Transcription
SP_1344	0.00209891	4.55E-06	192	139	conserved hypothetical protein	Signal transduction mechanisms
SP_0920; nspC	0.00220256	5.64E-05	74	51	carboxynorspermidine decarboxylase	Amino acid transport and metabolism
SP_0184	0.002298198	0.010837257	48	28	hypothetical protein	Function unknown
SP_0988; glmU	0.002304138	0.026471193	38	34	UDP-N-acetylglucosamine pyrophosphorylase	Cell wall/membrane/envelope biogenesis
SP_1067	0.002317923	0.001604033	40	39	putative cell division protein FtsW	Cell cycle control, cell division, chromosome partitioning
SP_1341	0.002382757	0.018432833	37	28	putative ABC transport system ATP-	Defense mechanisms
SP_1774	0.002440517	0.046405862	23	20	putative transcriptional regulator	Transcription
SP_0825; folD	0.002528829	0.001031481	26	22	dehydrogenase/methenyltetrahydrofolate cyclohydrolase	Nucleotide transport and metabolism
SP_1159	0.002532007	0.000269913	42	33	integrase/recombinase, phage integrase family	Cell cycle control, cell division, chromosome partitioning
SP_2075	0.0026384	8.50E-06	90	67	ATP-binding cassette, subfamily B,	Defense mechanisms
SP_1472	0.002738596	0.019055845	53	41	putative oxidoreductase	Function unknown
SP_1331	0.002764977	0.000449012	56	42	putative phosphosugar-binding	Transcription
SP_0606	0.002910556	0.009278222	72	53	putative oxidoreductase	Inorganic ion transport and metabolism
SP_1722	0.002918106	0.002280768	84	71	PTS system IIABC components	Carbohydrate transport and metabolism
SP_0056; purB	0.002964613	0.002810176	31	27	adenylosuccinate lyase	Nucleotide transport and metabolism
SP_1435	0.00340486	0.004702366	120	89	ABC transporter, ATP-binding protein	Defense mechanisms
SP_1438	0.003441197	0.001617803	89	61	ABC transporter, ATP-binding protein	Inorganic ion transport and metabolism
SP_0032; polA	0.003523194	2.62E-06	82	67	DNA polymerase I	Replication, recombination and repair
SP_1689	0.003594739	0.000583071	66	43	ABC transporter, permease protein	Inorganic ion transport and metabolism
SP_1869	0.003594787	0.019173312	59	39	iron-compound ABC transporter,	Inorganic ion transport and metabolism
SP_1294; crcB-1	0.00362266	3.75E-06	32	22	crcB protein fluride ion transport	Cell cycle control, cell division, chromosome partitioning
SP_2170; adcB	0.003693916	0.024072001	25	18	zinc ABC transporter, permease	Inorganic ion transport and metabolism
SP_0457; bacA	0.003907068	0.007436246	31	26	bacitracin resistance protein	Defense mechanisms
SP_2093	0.00396087	0.028556533	88	57	hypothetical protein	Function unknown
SP_2012; gap	0.004324867	0.00797438	22	19	glyceraldehyde 3-phosphate	Carbohydrate transport and metabolism
SP_1412; lgt	0.004571736	0.00469309	39	32	prolipoprotein diacylglyceryl transferase	Cell wall/membrane/envelope biogenesis
SP_1001	0.004615929	0.000300807	71	51	amino acid permease family protein	Amino acid transport and metabolism
SP_2231	0.004711046	0.000838861	153	115	putative ABC transporter, permease protein	Cell wall/membrane/envelope biogenesis
SP_2106	0.004877391	0.003386664	87	74	glycogen phosphorylase	Carbohydrate transport and metabolism
SP_1575	0.004898818	0.010847229	28	23	DNA replication protein	Replication, recombination and repair
SP_2192	0.004947018	4.36E-05	123	85	sensor histidine kinase	Signal transduction mechanisms
SP_1202; recN	0.004986126	0.000407477	68	53	DNA repair protein RecN	Replication, recombination and repair
SP_1222	0.005142161	0.019373801	92	74	type II restriction enzyme	Defense mechanisms
SP_0837	0.005146892	0.001621175	33	24	putative DNA topology modulation	Nucleotide transport and metabolism
SP_0153	0.005334332	0.033183659	28	22	putative membrane protein	Function unknown
SP_0251	0.00552636	0.000160956	182	118	putative formate acetyltransferase	Energy production and conversion
SP_1317; ntpA	0.005553887	0.036679786	111	69	V/A-type H+/Na+-transporting	Energy production and conversion
SP_1617	0.005724815	0.001373891	71	53	PTS system, fructose-specific IIC	Carbohydrate transport and metabolism
SP_1220; ldh	0.00581072	0.022112015	29	25	L-lactate dehydrogenase	Energy production and conversion
SP_0870	0.005874933	0.001198815	22	15	nitrogen fixation protein NifU and	Energy production and conversion
SP_0098	0.005931463	0.03568293	50	35	hypothetical protein	Function unknown
SP_0379	0.005994465	0.004756524	77	46	conserved hypothetical protein	Amino acid transport and metabolism, Carbohydrate transport and metabolism, Inorganic ion transport an
SP_0682	0.006026063	0.00089534	14	9	hypothetical protein	Function unknown
SP_1906; groEL	0.006189003	0.023519121	52	41	groEL; chaperonin, 60 kDa	Post-translational modification, protein turnover, and chaperones
SP_0672; hflX	0.006432674	2.22E-05	54	35	GTP-binding protein HflX	Post-translational modification, protein turnover, and chaperones
SP_0057; strH	0.006524184	0.002914485	210	144	beta-N-acetylhexosaminidase	Carbohydrate transport and metabolism
SP_0881; thiI	0.006633996	0.001324574	62	39	thiazole biosynthesis protein ThiI	Coenzyme transport and metabolism
SP_1040	0.006649688	1.64E-05	133	93	site-specific recombinase, resolvase	Replication, recombination and repair
SP_0462	0.007000639	0.000458804	156	110	cell wall surface anchor family protein	Cell wall/membrane/envelope biogenesis
SP_2239	0.007017314	0.000880407	63	49	serine protease htrA	Post-translational modification, protein turnover, and chaperones
SP_0892	0.007116942	3.92E-05	194	139	type I restriction enzyme, R subunit	Replication, recombination and repair
SP_0394	0.007253059	0.029189882	83	70	PTS system, mannitol-specific IIBC	Carbohydrate transport and metabolism

SP_1923; ply	0.007430822	0.00039954	74	52	ply; pneumolysin	Function unknown
SP_0468	0.007439853	0.036745299	38	31	putative sortase	Cell wall/membrane/envelope biogenesis
SP_0658; ccdA-1	0.007481676	2.25E-05	44	33	cytochrome c-type biogenesis protein CcdA	Post-translational modification, protein turnover, and chaperones
SP_2190; cbpA	0.007695521	0.000258659	80	60	choline binding protein A	Function unknown
SP_1056	0.007742429	0.040064931	79	62	Tn5252, relaxase	Intracellular trafficking, secretion, and vesicular transport
SP_1779	0.007835459	0.021978835	27	23	hypothetical protein	Function unknown
SP_1008; pepT	0.00804388	0.011206783	38	33	pepT; peptidase t	Amino acid transport and metabolism
SP_0166	0.008047975	9.45E-06	79	58	pyridoxal-dependent decarboxylase,	Amino acid transport and metabolism
SP_1246	0.008089694	0.000155017	39	30	Cof family protein	Secondary metabolites biosynthesis, transport, and catabolism
SP_0293	0.00813755	0.005459746	27	18	hypothetical protein	Function unknown
SP_1479; pgdA	0.008628615	0.047855137	63	52	pgdA; peptidoglycan N-	Carbohydrate transport and metabolism
SP_0927	0.008963067	0.006079935	35	30	transcriptional regulator, LysR family	Transcription
SP_2186; gplK	0.009010321	2.58E-05	93	66	gplK; glycerol kinase	Energy production and conversion
SP_1407	0.009198194	4.38E-06	37	28	(GenBank) hydrolase, haloacid dehalogenase-like family	Carbohydrate transport and metabolism
SP_1840	0.009290744	0.013289823	75	56	ABC transporter, ATP-binding/permease protein	Defense mechanisms
SP_1990	0.009545717	0.038196202	17	14	ribonuclease | (GenBank) primase-	Replication, recombination and repair
SP_1221	0.009549124	0.00735197	151	112	putative type II restriction	Replication, recombination and repair
SP_0762; metK	0.009774856	0.028893491	41	36	S-adenosylmethionine synthetase	Coenzyme transport and metabolism
SP_0250	0.009822205	0.034551936	95	66	component | PTS system, IIC component	Carbohydrate transport and metabolism
SP_0318	0.010284083	0.039329116	35	30	carbohydrate kinase, PfkB family	Coenzyme transport and metabolism
SP_2182	0.010297775	0.002265176	36	23	hypothetical protein	Function unknown
SP_0181	0.010534257	0.000742437	60	35	conserved hypothetical protein	Defense mechanisms
SP_1598	0.010692071	0.007429355	29	24	putative phosphomethylpyrimidine	Coenzyme transport and metabolism
SP_1687; nanB	0.010997615	2.36E-05	159	116	sialidase-1 | (GenBank) nanB;	Carbohydrate transport and metabolism
SP_0351; cps4F	0.011711768	0.006481642	67	51	capsular polysaccharide biosynthesis protein Cps4F	Cell wall/membrane/envelope biogenesis
SP_0703	0.011720843	0.022339964	26	20	hypothetical protein	Function unknown
SP_1586	0.011746085	0.015565242	61	48	ATP-dependent RNA helicase DeaD	Replication, recombination and repair
SP_1978; lysA	0.011779376	0.001582205	48	39	diaminopimelate decarboxylase	Amino acid transport and metabolism
SP_1652	0.012208541	0.003875907	149	113	putative ABC transport system permease protein (AMP)	Secondary metabolites biosynthesis, transport, and catabolism
SP_0641	0.01223566	4.59E-10	406	301	serine protease, subtilase family (prtA)	Post-translational modification, protein turnover, and chaperones
SP_1950	0.012321307	0.000428656	226	167	putative bacteriocin formation protein	Defense mechanisms
SP_1283; htpX	0.01251853	0.01076863	44	27	htpX; heat shock protein HtpX	Post-translational modification, protein turnover, and chaperones
SP_2145	0.012671644	0.010657397	92	69	cell wall surface anchor family	Carbohydrate transport and metabolism
SP_1483	0.012793484	0.022977308	42	36	ATP-dependent RNA helicase,	Replication, recombination and repair
SP_0312	0.01311958	0.000364936	136	88	glycosyl hydrolase, family 31	Carbohydrate transport and metabolism
SP_0689; murG	0.01315293	0.014107901	25	22	acetylmuramyl-(pentapeptide) pyrophosphoryl-undecaprenol N-	Cell wall/membrane/envelope biogenesis
SP_0695	0.013168646	0.000514489	65	48	HesA/MoeB/ThiF family protein	Coenzyme transport and metabolism
SP_1032	0.013430341	0.002418062	85	67	ron-compound ABC transporter, iron	Inorganic ion transport and metabolism
SP_0360; cps4L	0.01400452	0.021716239	68	53	UDP-N-acetylglucosamine 2-epimerase	Cell wall/membrane/envelope biogenesis
SP_0922	0.0140266	1.74E-05	50	36	carbon-nitrogen hydrolase family	Coenzyme transport and metabolism
SP_0478; lacE-1	0.014238031	1.10E-07	125	85	PTS system, lactose-specific IIBC	Carbohydrate transport and metabolism
SP_1793	0.014296069	0.0181452	56	39	hypothetical protein	No orthologs found
SP_1436	0.014466515	0.015175401	49	39	energy-coupling factor transport system substrate-specific component	Function unknown
SP_0445; ilvB	0.014661332	0.037496104	56	46	acetolactate synthase, large subunit, biosynthetic type	Amino acid transport and metabolism, Coenzyme transport and metabolism
SP_0320	0.014712282	0.000402688	27	25	oxidoreductase, short chain dehydrogenase/reductase family	Lipid transport and metabolism, Secondary metabolites biosynthesis, transport, and catabolism
SP_1911	0.014734929	0.005581243	17	10	putative thioredoxin	Post-translational modification, protein turnover, and chaperones
SP_1342	0.014873134	0.003822288	138	97	drug efflux ABC transporter, ATP-	Defense mechanisms
SP_2169; adcA	0.015089466	0.000128976	82	61	adcA; zinc ABC transporter, zinc-	Inorganic ion transport and metabolism
SP_2201; cbpD	0.015154961	0.034785017	56	44	cbpD; choline binding protein D	Function unknown
SP_1433	0.015296835	0.042647893	88	60	transcriptional regulator, AraC family	Transcription
SP_1357	0.015557979	3.01E-06	68	53	ABC transporter, ATP-	Amino acid transport and metabolism
SP_0908	0.015570744	0.001969944	88	70	protein Tex | (GenBank) putative	Transcription
SP_1845; exoA	0.015672945	0.000321665	26	20	exoA; exodeoxyribonuclease	Replication, recombination and repair
SP_1862	0.015694019	0.000993333	44	23	hypothetical protein	Function unknown
SP_2127	0.015991854	0.013322325	78	44	transketolase, C-terminal subunit	Carbohydrate transport and metabolism
SP_0718; thiE-1	0.016238066	0.009740231	53	33	thiamine-phosphate	Coenzyme transport and metabolism
SP_2168	0.016505348	0.002616516	55	39	putative fucose operon repressor	Transcription
SP_0785	0.016637184	0.020942731	47	30	HlyD family secretion protein	Cell wall/membrane/envelope biogenesis
SP_2227; recF	0.016899591	0.023375614	42	37	DNA replication and repair protein	Replication, recombination and repair
SP_0772	0.017158892	0.015378296	14	11	hypothetical protein	Function unknown
SP_2228; guaB	0.017620938	0.006359796	53	43	inosine-5'-monophosphate	Nucleotide transport and metabolism
SP_0240	0.017743864	0.000519906	19	17	phosphoglycerate mutase family	Carbohydrate transport and metabolism
SP_1604	0.017873027	0.000952089	32	24	hypothetical protein	Function unknown
SP_0729	0.01796614	0.021816956	88	70	Cu+-exporting ATPase	Inorganic ion transport and metabolism
SP_0671; miaA	0.019533447	0.003166489	29	24	tRNA isopentenylpyrophosphate transferase	Translation, ribosomal structure and biogenesis

SP_2120	0.020395568	0.002853924	57	36	hypothetical protein	Function unknown
SP_1175	0.020435082	0.036616382	121	87	conserved domain protein	Function unknown
SP_1871	0.020871476	0.010886815	14	13	iron-compound ABC transporter, ATP-	Inorganic ion transport and metabolism
SP_1400	0.021334218	0.00036004	52	32	putative phosphate ABC transporter,	Inorganic ion transport and metabolism
SP_1715	0.02142297	0.002462396	90	70	ABC transporter, ATP-binding protein	Inorganic ion transport and metabolism
SP_0627	0.021688058	0.020227881	50	31	conserved hypothetical protein	Carbohydrate transport and metabolism
SP_2087; pstB	0.021976245	0.041858399	32	27	phosphate ABC transporter, ATP-	Inorganic ion transport and metabolism
SP_1320; ntpE	0.022488963	0.002659233	36	24	v-type sodium ATP synthase, subunit	Energy production and conversion
SP_2144	0.022521704	0.013293113	55	45	conserved hypothetical protein	Carbohydrate transport and metabolism
SP_1234	0.022648326	0.029573876	37	23	transcriptional regulator, biotin	Transcription
SP_1061	0.023102226	0.000301724	93	69	putative protein kinase	Signal transduction mechanisms
SP_1775	0.02317772	0.0131502	31	16	conserved domain protein	Function unknown
SP_1765	0.023461008	0.000320101	87	67	glycosyl transferase, family 8	Cell wall/membrane/envelope biogenesis
SP_1431	0.023644415	0.028870123	67	53	putative type II DNA modification	Replication, recombination and repair
SP_1818	0.023825294	9.29E-06	17	9	hypothetical protein	No orthologs found
SP_0137	0.023944139	0.01618665	128	101	ABC transporter, ATP-binding protein	Amino acid transport and metabolism
SP_1645; relA	0.023969168	0.000751471	98	69	relA; GTP pyrophosphokinase	Transcription, Signal transduction mechanisms
SP_1280	0.024201742	0.040055166	23	21	conserved hypothetical protein	Transcription
SP_0546; blpZ;	0.024572351	0.01546026	13	9	blpZ; BlpZ protein, fusion	Function unknown
SP_1662; ylmH	0.024905854	0.02425892	27	21	ylmH protein	Translation, ribosomal structure and biogenesis
SP_0573	0.025063499	0.045619071	7	3	hypothetical protein	No orthologs found
SP_2082	0.025747041	0.005755258	17	15	family, alkaline phosphatase synthesis response regulator PhoP |	Signal transduction mechanisms
SP_0397; mtlD	0.025840017	0.000302917	63	48	mannitol-1-phosphate 5-	Carbohydrate transport and metabolism
SP_1563	0.025904281	0.004242853	22	19	pyridine nucleotide-disulphide	Energy production and conversion
SP_1702; secA-1	0.026197026	0.001194043	62	56	preprotein translocase, SecA subunit	Intracellular trafficking, secretion, and vesicular transport
SP_1544; aspC	0.026482654	0.039976919	45	35	aspartate aminotransferase	Amino acid transport and metabolism
SP_1063	0.028283885	0.046488604	57	48	ABC-2 transporter, permease protein	Defense mechanisms
SP_0150	0.028707352	0.000247404	62	43	peptidase, M20/M25/M40 family	Amino acid transport and metabolism
SP_1356	0.029039972	0.027295322	46	44	amidohydrolase family protein	Nucleotide transport and metabolism
SP_1050	0.029121145	0.000617284	20	15	antitoxin PezA | (GenBank) putative transcriptional regulator	Transcription
SP_1263; topA	0.029400682	0.013675354	57	50	topA; DNA topoisomerase I	Replication, recombination and repair
SP_1991	0.029614098	0.023976684	40	24	TatD DNase family protein | putative	Replication, recombination and repair
SP_0480	0.029974226	3.65E-05	64	48	potassium uptake protein, Trk family	Inorganic ion transport and metabolism
SP_0069; cbpI	0.030010513	0.00408953	61	37	choline binding protein	Function unknown
SP_0060	0.030475424	0.003535371	88	68	beta-galactosidase | glycosyl	Carbohydrate transport and metabolism
SP_0229; rplO	0.03108821	0.040330262	10	8	ribosomal protein L15	Translation, ribosomal structure and biogenesis
SP_1772	0.031170968	0.010724695	84	68	cell wall surface anchor family protein	Function unknown
SP_1243; zwf	0.031226331	0.003327536	58	44	glucose-6-phosphate 1-	Carbohydrate transport and metabolism
SP_2036	0.031696216	0.001043251	47	27	PTS system, IIA component	Carbohydrate transport and metabolism
SP_0585; metE	0.031832891	0.002071892	123	78	5-methyltetrahydropteroyltriglutamate--homocysteine methyltransferase	Amino acid transport and metabolism
SP_0061	0.031973192	0.006747456	23	14	PTS system, IIB component	Carbohydrate transport and metabolism
SP_0894; pepX	0.032434046	0.014046941	112	80	X-pro dipeptidyl-peptidase	Defense mechanisms
SP_0871	0.032601775	6.62E-05	63	57	Fe-S cluster assembly protein SufB	Post-translational modification, protein turnover, and chaperones
SP_0751; livM	0.032725063	0.0255206	53	39	branched-chain amino acid transport	Amino acid transport and metabolism
SP_0065; agaS	0.032999636	0.000741059	76	46	sugar isomerase domain protein AgaS	Cell wall/membrane/envelope biogenesis
SP_0025	0.033025067	0.03425237	20	14	hypothetical protein	No orthologs found
SP_0101	0.033595892	5.65E-05	80	52	putative transporter	Inorganic ion transport and metabolism
SP_1560	0.034268732	0.00034267	33	24	conserved hypothetical protein	Post-translational modification, protein turnover, and chaperones
SP_1240	0.034357057	0.000180501	30	25	conserved hypothetical protein	Defense mechanisms
SP_1212; truB	0.034666894	0.035913329	42	26	; tRNA pseudouridine synthase B	Translation, ribosomal structure and biogenesis
SP_0043; comB	0.034700505	0.012066145	71	48	competence factor transport protein ComB	Cell wall/membrane/envelope biogenesis
SP_1014; dapA	0.034960467	0.022790656	55	43	dihydrodipicolinate synthase	Amino acid transport and metabolism
SP_1306; gdhA	0.035798907	0.001834463	69	48	NADP-specific glutamate	Amino acid transport and metabolism
SP_1021; hemK	0.035938169	0.01624595	41	36	release factor glutamine methyltransferase	Translation, ribosomal structure and biogenesis
SP_1394	0.036206135	0.003581419	44	27	amino acid ABC transporter, amino	Inorganic ion transport and metabolism
SP_2055; Adr	0.036714301	0.293686896	35	24	alcohol dehydrogenase, zinc-	Energy production and conversion
SP_1083	0.037003072	0.002384272	44	32	conserved hypothetical protein	Transcription
SP_0577	0.037685426	0.004199406	104	84	PTS system, beta-glucoside-specific	Carbohydrate transport and metabolism
SP_1143	0.037732489	0.00764027	24	17	conserved hypothetical protein	Transcription
SP_1668	0.037758911	0.04787245	35	27	hypothetical protein	Function unknown
SP_0400; tig	0.037984653	0.012213689	43	32	trigger factor	Cell cycle control, cell division, chromosome partitioning
SP_1088; radC	0.038040116	0.000998223	24	18	DNA repair protein RadC	Replication, recombination and repair
SP_0374	0.038356529	0.043024497	57	47	mid-cell-anchored protein Z	Cell cycle control, cell division, chromosome partitioning
SP_1453	0.039165214	0.005693396	46	25	poly-gamma-glutamate synthesis protein (capsule biosynthesis protein)	Cell wall/membrane/envelope biogenesis
SP_0306	0.039446901	0.008449249	84	61	lichenan operon transcriptional	Transcription
SP_2143	0.03971822	0.000294554	94	67	alpha-mannosidase	Carbohydrate transport and metabolism
SP_1752	0.039928984	0.024172394	24	19	putative mechanosensitive ion channel	Cell wall/membrane/envelope biogenesis

SP_0194	0.040311842	0.011941424	9	7	conserved hypothetical protein	Function unknown
SP_0616; fibB	0.04097539	0.006665097	59	53	alanine adding enzyme | (GenBank) fibB; beta-lactam resistance factor	Defense mechanisms
SP_0077	0.041557894	0.004791098	7	5	hypothetical protein	Function unknown
SP_2158; fucI	0.042077751	0.002652512	100	65	L-fucose isomerase	Carbohydrate transport and metabolism
SP_0722; tenA	0.042241489	0.000590715	68	36	thiaminase (transcriptional activator	Transcription
SP_0833	0.042673756	0.018687771	29	25	hypothetical protein	Function unknown
SP_0405	0.043207291	0.00099568	30	24	conserved hypothetical protein	Function unknown
SP_1551	0.044462649	0.000750776	76	66	Ca2+-transporting ATPase	Inorganic ion transport and metabolism
SP_0567	0.044700634	0.029302586	40	28	conserved domain protein	Nucleotide transport and metabolism
SP_2056; nagA	0.044705085	0.012127935	83	55	N-acetylglucosamine-6-phosphate	Carbohydrate transport and metabolism
SP_0647	0.044771262	0.002191097	67	53	PTS system, galactose-specific IIC	Carbohydrate transport and metabolism
SP_0799; ciaH	0.045607306	0.00313974	60	53	two-component system, OmpR	Signal transduction mechanisms
SP_1988	0.045930843	0.001558823	98	72	permease protein | (GenBank) putative immunity protein	Defense mechanisms
SP_1242	0.046415303	0.014270106	27	23	amino acid ABC transporter, ATP-	Amino acid transport and metabolism
SP_1749	0.046786029	0.025483512	27	24	30S ribosome assembly GTPase |	Function unknown
SP_1971	0.046788458	0.022771553	19	13	hypothetical protein	Function unknown
SP_1754	0.047375889	0.004080247	47	33	conserved hypothetical protein	Function unknown
SP_1832	0.047438851	0.007579274	29	21	hypothetical protein	Function unknown
SP_1930	0.048750085	0.002071426	12	10	hypothetical protein	Function unknown
SP_1981; ccs50	0.048895703	0.000832798	35	29	(GenBank) ccs50; competence-induced protein Ccs50	Function unknown
SP_0518	0.049202841	0.008026713	14	9	hypothetical protein	No orthologs found
SP_2151; arcC	0.049769238	0.044333763	33	26	arcC; carbamate kinase	Amino acid transport and metabolism

Supplementary Table 2: Effect of Immunization on Transmission of Strain T4S[#]

	Uninfected contacts	Infected contacts	Transmission rate
Mock	3	11	78.6%
Immunized	10	12	54.5%[*]

[#] Dams were immunized and boosted with all five candidate antigens (StkP,PenA,PgdA,HtrA, and LytD) in adjuvant or adjuvant alone (mock)

[*] *P*=0.175 (Fisher's Exact Test)

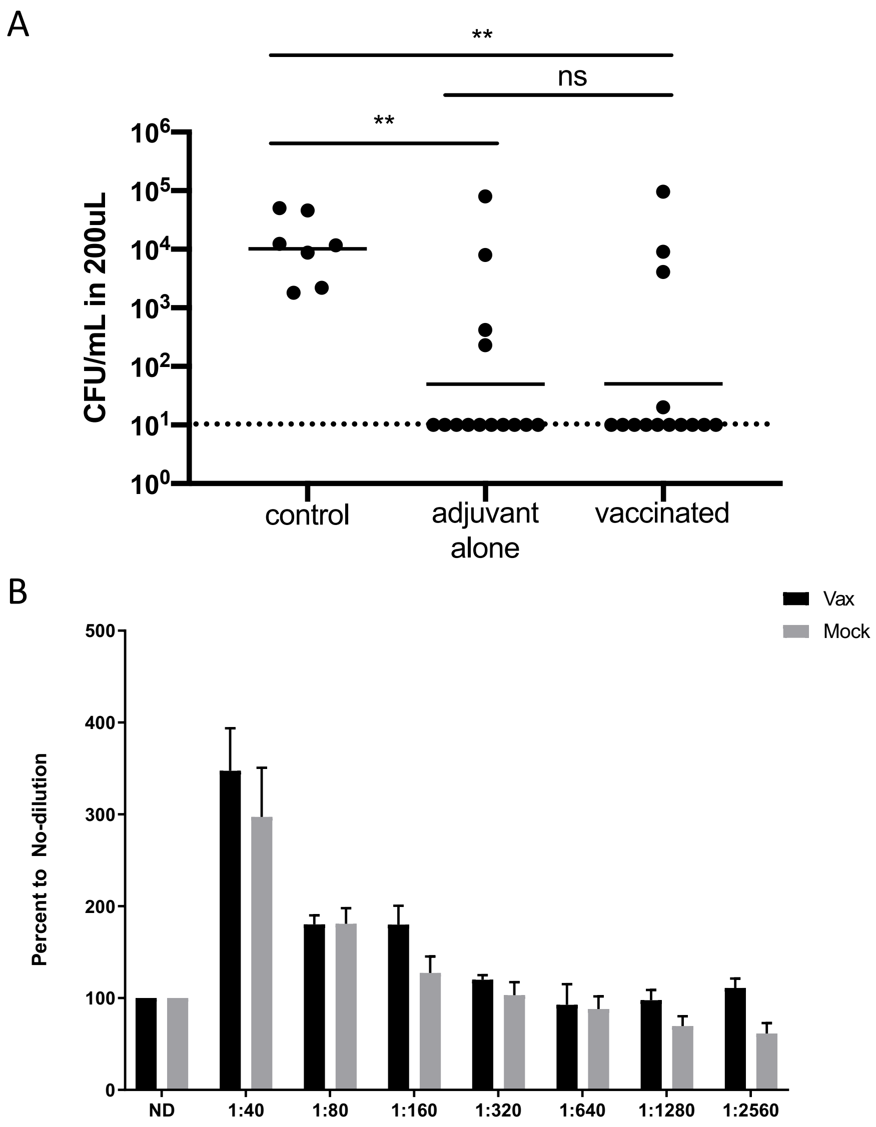

Supplementary Figure 1. Effect of immunization on colonization of adult mice (A). Mice were vaccinated with two doses of the five antigen combination with adjuvant and compared to adjuvant alone and untreated age-match controls. 5 days post-inoculation with a type 23F isolate colonization density was determined in upper respiratory tract lavages. Each symbol represents an individual mouse with the median value and limit of detection shown. ***P*<0.01(Kruskal Wallis with Dunn's post-test). ns, non-significant. Effect of immune sera on growth (B). Strain T4S was growth in tryptic soy broth with pooled sera at the dilution indicated from adult mice immunized with the five antigen combination (vax) and compared to adjuvant only controls (mock). Growth was assessed after three hours by measuring the OD_{620} and expressed relative to no sera controls $\pm$ S.D.

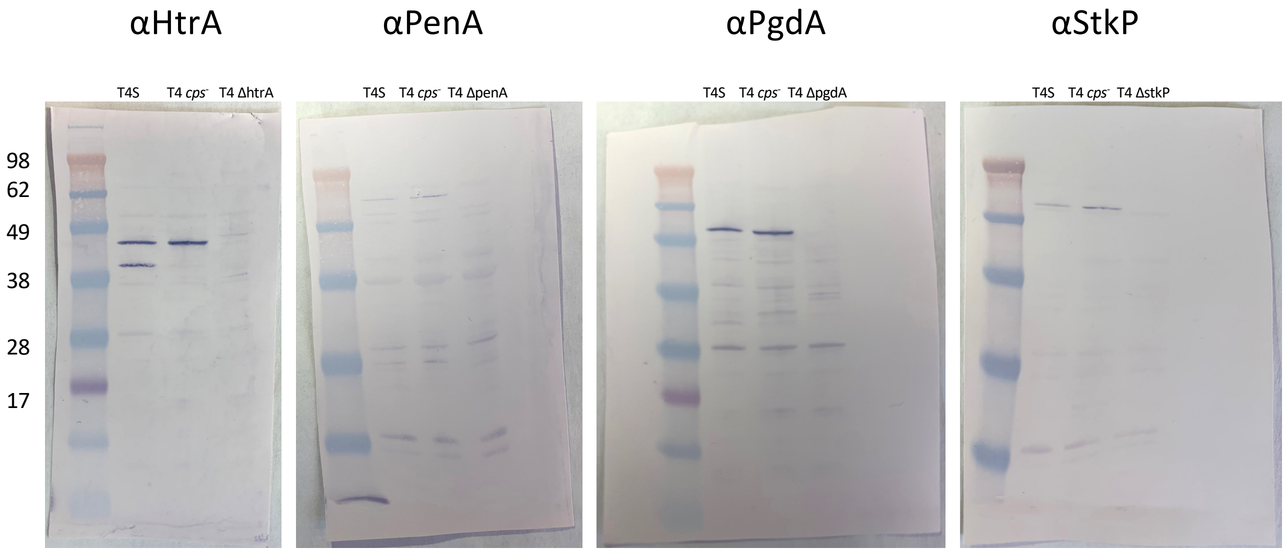

Supplementary Figure 2. Western analysis showing the entire blot used in constructing Figure 3d. Pooled sera (n=5) from adult mice immunized with the individual antigen shown was used in Western analysis on whole cell lysates of strain T4S, the unencapsulated mutant T4cps- or the mutant construct corresponding to the sera tested. Size markers in kilodaltons.

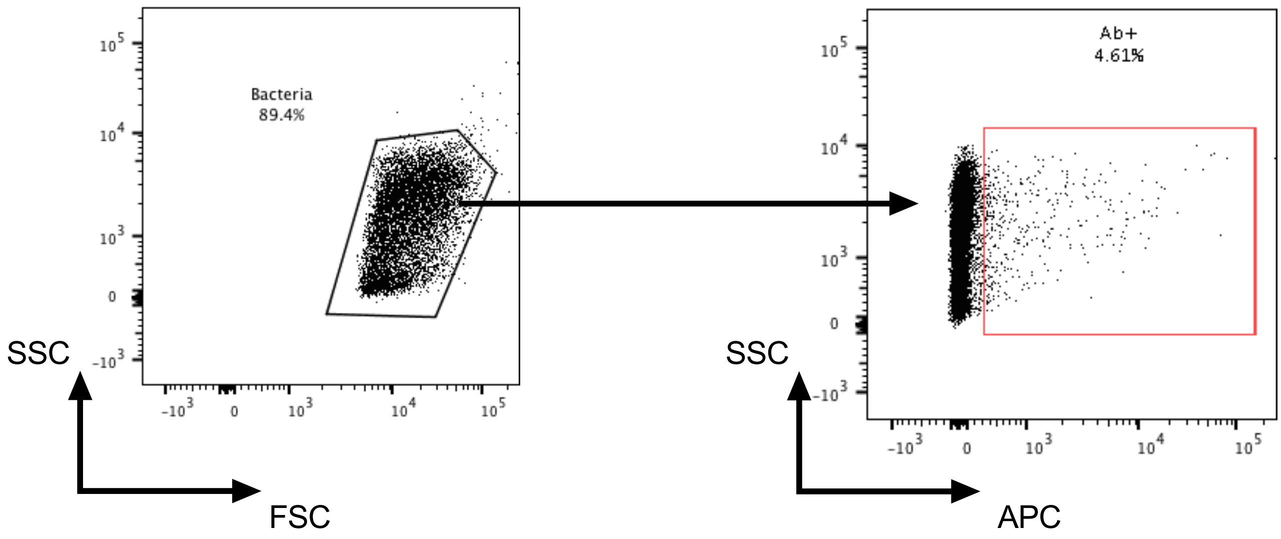

Supplementary Figure 3: Gating strategy used for flow cytometry. This strategy was used for detection of antibody binding to pneumococcal surface antigens in Figure 3E.
